# Two-year longitudinal health-related fitness, anthropometry and body composition status amongst adolescents in Tlokwe Municipality: The PAHL Study

**DOI:** 10.4102/phcfm.v7i1.896

**Published:** 2015-10-30

**Authors:** Oluwatoyi O. Toriola, Makama A. Monyeki, Abel L. Toriola

**Affiliations:** 1 Physical Activity, Sport and Recreation Focus Area, North-West University, Potchefstroom Campus, South Africa; 2 2Department of Sport, Rehabilitation and Dental Sciences, Tshwane University of Technology, South Africa

## Abstract

**Aim:**

To evaluate a two-year longitudinal development of health-related fitness, anthropometry and body composition status amongst adolescents in Tlokwe Municipality, Potchefstroom, South Africa.

**Setting:**

A total of 283 high-school learners (111 boys and 172 girls) of ages 14 and 15 years who were part of the ongoing Physical Activity and Health Longitudinal Study (PAHLS) participated in the study. For the purpose of the present study, data collected for 2011 and 2012 for anthropometric, body composition and health-related physical fitness were used.

**Results:**

Body mass index (BMI) classification of boys and girls for 2011 and 2012 showed that 24.3% of them were underweight compared with 21% in 2012. In 2011, 50% of boys and girls had normal body weight compared with 52% in 2012, whilst 25.5% of the total group of participants were overweight compared with 27% in 2012. Both boys and girls showed significant increases of 5.9% in body fat (BF) and 3.2 kg in body weight over two years’ measurements, respectively. Regarding health-related fitness (i.e BAH), boys showed an increase of 14.8 seconds whilst girls gained 9.6 seconds. Significant decreases were found for sit-ups in both boys and girls. A significant VO_2_max increase of 2.9 mL/kg/min. was found in boys over the time period. A non-significant decrease of −0.5 mL/kg/min. was observed in girls. Regression coefficients showed that changes in BMI were inversely associated with those in health-related physical fitness. The changes in percentage BF were negatively associated with standing broad jump (SBJ), bent arm hang (BAH) and VO_2_ max in both boys and girls. A low significant positive association was found between changes in waist-to-height ratio (WHtR) and SBJ in both genders, whilst inverse low associations were found between WHtR and BAH in girls and for VO_2_max in both genders.

**Conclusion:**

Changes in BMI, %BF and WHtR were negatively associated with strength and running performances in the participating children. The relative increase in overweight, especially in girls, negatively affected their endurance running and static strength performances. The health implications of the observed findings are discussed and recommendations offered for physical activity intervention in school physical education (PE) programmes.

## Abstrait

**Etude longitudinale de deux ans de la condition physique liée à la santé, de l’anthropométrie et de l’état de la composition corporelle chez des adolescents de la municipalité de Tlokwe: Etude ELAPS.**

**Objectifs:**

Evaluer pendant deux ans le développement longitudinal de la condition physique liée à la santé, de l’anthropométrie et de l’état de la composition corporelle chez les adolescents de la municipalité de Tlokwe, à Potchefstroom, en Afrique du Sud.

**Lieu:**

283 élèves du secondaire (111 garçons et 172 filles) de 14–15 ans faisant partie de l’Etude longitudinale de l’Activité physique et de la Santé (ELAPS) ont participé à l’étude. Pour cette étude on a utilisé les données collectées en 2011 et 2012 pour l’anthropométrie, la composition corporelle et la condition physique liée à la santé.

**Résultats:**

La classification de l’index de masse corporelle (IMC) des garçons et des filles en 2011 et 2012 a montré que 24.3% d’entre eux avaient un poids insuffisant comparé à 21% en 2012. En 2011, 50% des garçons et des filles avaient un poids normal comparé à 52% en 2012, alors que 25.5% du groupe participant étaient en surpoids comparé à 27% en 2012. Les garçons tout comme les filles montraient une augmentation significative de 5.9% de graisse corporelle (GC) et 3.2 kg de masse corporelle sur deux ans, respectivement. En ce qui concerne la condition physique liée à la santé (i.e. BAH), les garçons avaient gagné 14.8 secondes et les filles 9.6 secondes. On a trouvé une diminution significative pour les redressements assis chez les garçons comme chez les filles. On a remarqué une augmentation VO_2_max de 2.9 mL/kg/min. chez les garçons pendant cette période. On a noté une diminution peu importante de −0.5 mL/kg/min. chez les filles. Les coefficients de régression ont montré que les changements en IMC étaient inversement associés à ceux de la condition physique liée à la santé. Les changements dans le pourcentage de GC étaient liés négativement avec le saut en longueur sans élan (SLSE), la suspension à bras pliés (SBP) et le VO_2_max chez les garçons et les filles. On a trouvé un rapport significatif entre les changements du rapport Tour de taille sur Taille (RTtT) et le SLSE chez les deux sexes, alors qu’il y avait un faible rapport inverse entre le RTtT et la SBP chez les filles et pour le VO_2_max chez les deux sexes.

**Conclusion:**

Les changements dans l’IMC, le %GC et le RTtT étaient associés négativement avec la force et la performance à la course des enfants participants. L’augmentation relative de poids, surtout chez les filles, a affecté leur endurance à la course et leurs performances de force statique. Les implications pour la santé des résultats observés sont discutées et des recommandations sont proposées de plans d’activités physiques dans les programmes d’Education physique (EP) des écoles.

## Introduction

Childhood obesity has become a major public health concern in the new millennium^1^ and is one of the most important risk factors of non-communicable diseases in both developed and developing countries.^2^ The prevalence of underweight, overweight and obesity in youth has resulted in an increased mortality rate worldwide.^3,4^ Therefore, it is of vital importance to understand trends in the development of body weight disorders in the youth and the negative effect thereof on overall health.^5^

According to the World Health Organization (WHO), physical inactivity is one of the fastest growing risk factors of non-communicable diseases.^6^ For instance, the WHO reported that the number of children below the age of 5 who were overweight was estimated to be at least 42 million worldwide.^7^ Furthermore, obesity and overweight have been found to be major contributors to the risk factors of cardiovascular disease amongst children and adolescents.^8,9^ Measured in terms of body mass index (BMI) and percentage body fat (%BF), obesity has been found to be negatively associated with performance tasks in which the body is raised off the ground, such as standing broad jump (SBJ), and the tasks for which the body has to be lifted in space, such as bent arm hang (BAH) and sit-ups.^10^

From early childhood, a sedentary lifestyle and poor health-related fitness lead to undesirable health consequences,^11^ including imbalances in body composition. The increasing prevalence of childhood overweight and obesity over the past decades therefore raises concern.^11^ Amongst the detrimental consequences of childhood overweight on current and future health are that children and adolescents who are overweight have very low levels of fitness compared with those who are slimmer.^12,13^ Furthermore, overweight is found to be a leading factor associated with inactivity amongst children.^14^

Excessive consumption of food high in kilojoules, and physical inactivity, worsened by prolonged TV viewing, the use of social media and playing computer video games, have also been identified as major contributory factors to the high incidence of overweight and obesity amongst children and adolescents.^8^ Wiecha et al.^15^ reported that the prevalence of overweight children and adolescents has increased between 1976 and 1994 to 13%, is continuously increasing, and reached about 15.5% in 2000. Furthermore, it was revealed that most of the youths affected lived in developing nations. The rate at which children in this domain consume fatty food is alarming.^1^ The WHO stated that the excessive consumption of processed food leads to accumulation of body fat (BF) and a high BMI, causing overweight and finally leading to obesity.^16^

From an epidemiological standpoint, obesity is associated with physical inactivity and is assumed to be the outcome of several health risk factors during childhood and adolescence^17^; hence, the need for strategic interventions to stem the menace of overweight and physical inactivity in children is imperative.^5^

In view of the link between physical activity, physical fitness and body composition in children, it is important to have a comprehensive understanding of the relationship, using prospective research design in order to implement appropriate intervention strategies. However, very little epidemiological research exists that examines such longitudinal trends.^18,19^ Furthermore, few studies on the longitudinal relationship between health-related physical fitness, physical activity and body composition amongst South African children have been conducted.^20,21,22^ Therefore, the purpose of the present study was to evaluate the two-year longitudinal development of health-related fitness, anthropometry and body composition status amongst adolescents in Tlokwe Municipality, South Africa.

## Methods

### Design

The present research was part of the ongoing Physical Activity and Health Longitudinal Study (PAHLS),^5^ which commenced in 2010 following a mixed longitudinal survey designed to evaluate the development of physical activity (PA), determinants of health risk, and factors affecting participation in sport and recreational activities amongst 14-year-old high school learners in Tlokwe Municipality, an area within Dr Kenneth Kaunda District Municipality which is located near Potchefstroom, the capital city of North-West Province, South Africa. For the purpose of the present study, analyses of findings from the cross-sectional measurements of 2011 and measurements of 2012 are presented.

### Participants

A total of 283 high school learners (111 boys and 172 girls), aged 14 and 15 years, who were purposefully drawn from six secondary schools in Tlokwe Municipality, which granted permission to carry out the research, participated in the study. The participants provided demographic information regarding gender, race and school locale (i.e. town or township). The school-based locales comprised four schools from the town and four others in economically deprived township areas. The former group of schools (Potchefstroom town) thus comprised learners predominantly of high socioeconomic background, and the latter group of schools (Ikageng township) comprised learners predominantly of low socioeconomic background. The South African Department of Education categorises schools in quintiles (1 to 5) according to physical condition, facilities and crowding, and the relative poverty of the community around the schools.^23^ Of the 8 schools initially selected, 2 urban schools declined to participate in the study, without giving any reasons. Detailed information regarding the participants^5^ and methods of data collection have been published elsewhere.^8^

### Anthropometric measurements

The participants’ height, body weight, skinfold thickness (triceps and subscapular skinfolds), and waist circumference (WC) respectively were measured using the protocol of the International Society for the Advancement of Kinanthropometry (ISAK).^24^ Waist-to-height ratio (WHtR) and BMI were calculated as waist (cm)/height (cm) and mass/stature² (kg.m^−^²), respectively. Subsequently, age-specific BMI for children was used to determine the following categories: overweight, normal weight and underweight/thinness.^25^ Percentage body fat (%BF) for boys was: 2 = very low; 6–12 = low; 13–18 = optimal range; 19–25 moderately high; 26–31 = high; ≥ 32 very high; and %BF for girls was: 4–10 = very low; 11–15 = low; 15–24 = optimal range; 26–30 = moderately high; 31–35.5 = high; ≥ 35.5 = very high). % BF was calculated from skinfold measurements using Slaughter et al.'s^26^ equation which is internationally accepted for use in children and adolescents from different ethnic groups.

### Health-related physical fitness measurements

The participants’ health-related physical fitness status was determined by assessing their cardiorespiratory endurance, muscle strength and endurance, and flexibility, using standardised techniques.^27,28^ Cardiovascular endurance was assessed with the 20-metre multistage shuttle run test which is a valid measure of aerobic capacity in adolescents.^29^ The following health-related fitness test items were measured according to the EUROFIT^28^ test protocol: sit-and-reach (SAR) (a test of hamstring flexibility, expressed in centimetres); sit-up (SU) (a measure of abdominal strength and endurance, determined by the number of correctly performed SUs in 30 seconds); SBJ (a test of explosive strength of leg extensors measured in centimetres), and BAH (which measures functional arm and shoulder muscular endurance to exhaustion in seconds).

### Measurement procedures

Before data collection, permission to conduct the measurements was granted by the District Manager of the Department of Basic Education in Potchefstroom, North-West Province. Clearance was also received from the Ethics Committee of North-West University, Potchefstroom Campus (Ethics no: NWU-0058-01-A1). The participating schools were briefed about the purpose of the study, and informed consent forms were signed by the school authorities as well as the learners and their parents. To minimise loss of interest and fatigue amongst the participants and prevent disruption of teaching and learning activities at the schools, data were collected on days agreed to by the participating schools. Only the data of learners who were 14 and 15 years old at the time of testing were analysed.^5^

Before the anthropometric and health-related fitness measurements were carried out, a demographic questionnaire, under the supervision of the principal investigator, was completed. The physical and physiological variables were measured in the following order: anthropometry, health-related fitness, and gross and fine motor fitness. All anthropometric sites were measured twice by Level 2 ISAK-certified anthropometrists according to standardised procedures.

### Data analyses

The cross-sectional data on the high school learners’ health-related fitness, body composition and PA were analysed using descriptive statistics, such as means and standard deviations. Independent *t*-tests were computed to determine age and sex differences in the variables amongst the high school learners. A non-parametric paired *t*-test was used to examine significant differences between two outcome variables, and a chi-square test to evaluate significant differences. The effect size statistics reported as Cohen's *d* (standardised mean differences) and 95% confidence interval (CI) were calculated. Values of Cohen's *d* = 0.2, 0.5 and 0.8 were regarded as small, medium and large effects, respectively. To study the development over time of the body composition indicators and health-related physical fitness, Pearson's product moment correlation coefficients were calculated for the first and second measurements for all outcome variables. To further examine the relationships between the independent and dependent measures, linear regression analyses corrected for gender, race and locality were computed. If a significant interaction with gender was established, separate coefficients were reported for boys and girls separately. All data analyses were performed with the Statistical Package for the Social Sciences (SPSS), Version 20.0 2011. A probability level ≤ 0.05 was taken to indicate significance.

## Results

Table 1 presents the percentage distribution of the learners’ BMI categories by year of study (i.e. for both 2011 and 2012). Based on BMI classifications of boys and girls over the 2-year period, 24.3% of them were underweight, with a decrease of 3.3 (21%) in 2012. For 2011, 50% of the boys and girls were in the normal weight category, with an increase of 2% (52%) in 2012, whilst 25.5% of the total group of participants was overweight in 2011, thus yielding an overall increase of 1.5% (27%) in 2012.

**TABLE 1:** Percentage distribution of BMI categories for total group of participants by year of study.

BMI classification	2011 (boys and girls)		2012 (boys and girls)	
	Frequency	%	Frequency	%
Underweight (BMI between 17 and 18.5)	60	24.3	52	21
Normal weight (BMI between 18.5 and 25)	124	50.2	128	52
Overweight (BMI > 22.62 for boys and > 23.34 for girls)	63	25.5	66	27
**Total**	**247**	**100.0**	**246**	**100**

BMI, body mass index.

Table 2 comprises data on the percentage distribution of BMI by gender for the learners in 2011 and 2012. In the measurements for 2011, 23% of the boys were in the underweight category; a decrease of 3.5% in the incidence of underweight was found amongst them in the second measurement session in 2012. For girls, 25% were underweight in 2011, and in 2012 a decrease of 3.1% was found. Amongst the boys, those overweight showed an increase in incidence of 11.5%, whilst a decrease in incidence of 4.3% was found amongst the girls.

**TABLE 2:** Percentage distribution of BMI of participants by gender for 2011 and 2012.

BMI classification	2011				2012			
	**Boys**		**Girls**		**Boys**		**Girls**	
	Frequency	%	Frequency	%	Frequency	%	Frequency	%
Underweight (BMI between 17 and 18.5)	20	23.0	40	25.0	17	19.5	35	21.9
Normal weight (BMI between 18.5 and25)	49	56.3	75	46.9	42	48.3	86	53.8
Overweight (BMI > 22.62 for boys and > 23.34 for girls)	18	20.7	45	28.1	28	32.2	38	23.8
**Total**	**87**	**100.0**	**160**	**100**	**87**	**100.0**	**159**	**99.4**

BMI, body mass index.

Descriptive data by gender differences in the anthropometric and body composition characteristics (boys and girls in 2011 and 2012) are shown in Table 3. In 2011, the boys’ mean height was 160.7 ± 9.1 cm (*n*= 87) which reveals that they were not significantly taller than the mean height of 162.6 ± 9.4 cm (*n*= 87) (*p*> 0.117) noted in 2012. For the girls in 2012, results showed that they were taller (163.0 ± 9.43 cm; *n*= 159) than they were (157.8 ± 7.09 cm; *n* = 159) in 2011, but the difference was not significant (*p*> 0.608). Boys were slightly heavier (57.09 ± 14.17 kg; *n*= 87) in the first year than in the second year (57.02 ± 13.4 kg; *n*= 87), but the difference was also not statistically significant (*p*> 0.959). However, in the second year, girls were heavier (57.33 ± 14.0 kg; *n*= 159) than they were in the first year (54.11 ± 13.15 kg; *n*= 159), similarly yielding a non-significant difference (*p*> 0.992).

**TABLE 3:** Gender differences in the high school learners’ anthropometric and body composition characteristics.

Variables	Boys			Girls				**Cohen's *d***
	2011 (*n* = 87)	2012 (*n* = 86)		2011 (*n* = 159)		2012 (*n* = 158)		
	Mean ± SD	Mean ± SD	Average mean change over time and confidence intervals	Cohen's *d*	Mean ± SD	Mean ± SD	Mean differences	
Stature (cm)	160.7 ± 10.0	162.6 ± 9.4	1.9 (−7.42 to −1.06) †	0.65	157.8 ± 7.09	163.0 ± 9.43	5.2 (−7.16 to −3.39) †	0.67
Body mass (kg)	57.1 ± 14.17	57.0 ± 13.4	−0.1 (−4.10 to 4.25)	0.02	54.1 ± 13.15	57.3 ± 14.0	3.2 (−6.23 to −0.21)†	0.25
BMI (kg.m^−^2)	20.5 ± 3.81	21.7 ± 4.56	1.2 (−2.48 to 0.04)	0.69	21.6 ± 4.45	21.4 ± 4.30	−0.2 (−0.52 to 1.17)	0.03
%BF	14.44 ± 9.09	20.3 ± 9.36	5.9 (−8.41 to −3.11)†	0.89	26.4 ± 8.61	19.4 ± 9.68	−7 (4.87 to 9.10)†	0.89
WHtR (mm)	0.42 ± 0.04	0.43 ± 0.05	0.01(−0.02 to 0.01)	0.33	0.43 ± 0.05	0.42 ± 0.51	−0.01 (−0.00 to 0.02)†	0.47
SBJ (cm)	156.4 ± 26.2	162.5 ± 86.7	6.1 (−26.11 to 13.82)	0.48	165.7 ± 30.8	159.8 ± 54.7	−5.9 (−3.77 to 15.59)	0.09
BAH (x/sec.)	8.0 ± 9.38	22.8 ± 12.4	14.8 (−18.43 to −11.15)†	0.88	10.8 ± 12.1	20.4 ± 14.2	9.6 (−12.50 to -6.71)†	0.87
SU (x/sec.)	29.3 ± 10.7	13.9 ± 15.36	−15.4 (11.32 to 19.42)†	0.92	28.0 ± 10.46	17.0 ± 14.3	−11 (8.43 to 13.66)†	0.84
VO_2_max (mL/kg/min)	31.2 ± 7.75	34.2 ± 7.96	3 (−5.58 to −0.36)†	0.56	34.6 ± 8.33	34.1 ± 8.56	−0.5 (−1.58 to 2.61)	0.15
SAR (cm)	44.7 ± 8.64	45.8 ± 8.42	1.1 (−3.55 to 1.25)	0.10	45.6 ± 8.55	46.1 ± 8.76	0.5 (−2.33 to 1.38)	0.02

†, A significant change over time with p < 0.05.

SD, standard deviation; BMI, body mass index; %BF, percentage body fat; WHtR, waist-to-height ratio; SBJ, standing broad jump; BAH, bent arm hang; SU, sit-up; VO2max, maximal oxygen uptake; SAR, sit-and-reach.

Both series of measurements for boys showed a significant increase of 1.9 cm (95% CI −7.42 to −1.06 and Cohen's *d*= 0.63) in height, and 5.9% BF (95% CI −8.41 to −3.11 and Cohen's *d*= 0.89), over 2 years. Amongst the girls, a significant increase of 5.2 cm (95% CI −7.16 to −3.39 and Cohen's *d*= 0.67) in height and 3.2 kg (95% CI −6.23 to −0.21 and Cohen's *d* = 0.25) in body weight were found. Regarding health-related physical fitness, boys showed a significant increase of 14.8 seconds (95% CI −18.43 to −11.15 and Cohen's *d* = 0.88) in BAH and 3 mL/kg/min. (95% CI −5.58 to −0.36 and Cohen's *d* = 0.56) in VO_2_max. A significant decrease of 15.4 counts per second (95% CI 11.32 to 19.42 and Cohen's *d* = 0.88) was found in SUs in boys. For girls, a significant increase of 9.6 seconds (95% CI −12.50 to −6.71 and Cohen's *d* = 0.87) in BAH was found. Additionally, a significant decrease of 11 counts (95% CI 8.43 to 13.66 and Cohen's *d* = 0.85) was found for SUs in girls. A non-significant decrease of 5.9 cm (95% CI −3.77 to 15.59 and Cohen's *d* = 0.09) in SBJ and 0.5 mL/kg/min (95% CI −1.58 to 2.61 and Cohen's *d* = 0.15) in VO_2_max were observed (Table 4).

**TABLE 4:** Correlation coefficients for body composition variables over two annual measurements.

Variables	%BF2	Body mass2	BMI2	% BF2	∑ SKF2	WHtR2
% BF1	-	0.02	−0.02	−0.06	−0.06	−0.07
Body mass1	.02	-	0.86†	0.44†	0.55†	0.70†
BMI1	−.02	0.86†	-	0.72†	0.81†	0.88†
% BF1	−.06	0.44†	0.72†	-	0.96†	0.65†
∑SKF1	−.06	0.55†	0.81†	0.96†	-	0.75†
WHtR1	−.07	0.70†	0.88†	0.65†	0.75†	-

†, Correlation is significant at the 0.01 level (2-tailed).

%BF, percentage body fat; BMI, body mass index; ƩSKF, sum of skinfolds; WHtR, waist-to-height ratio.

Significantly high correlation coefficients were found for BMI and %BF, sum of skinfolds and WHtR at all measurements points for all participants (Table 5).

**TABLE 5:** Correlation coefficients for health-related fitness over two annual measurements.

Variables	SBJ2	SUP2	BAH2	SAR2	VO_2_max2
SBJ1	−0.02	0.07	0.02	0.001	0.02
BAH1	−0.03	0.11	−0.01	0.04	0.02
SUP1	0.04	0.09	0.10	−0.002	0.05
VO_2_max1	−0.05	0.10	−0.02	0.002	0.02
SAR1	−0.01	−0.06	0.04	0.13†	0.09

†, correlation is significant at the 0.05 level (2-tailed).

The results also showed low non-significant correlation coefficients between health-related physical fitness in the first and second measurements, except for a significant correlation found between SAR at first and second measurements.

Table 6 presents the age-adjusted regression coefficients for the relationship of changes in body composition and the health-related fitness parameters. The changes in BMI were inversely associated with health-related physical fitness. The changes in %BF were negatively associated with SBJ, BAH and VO_2_max in both boys and girls. A low significant positive association was found between changes in WHtR and SBJ in both genders, whilst low inverse associations were found between WHtR and BAH in girls and for VO_2_max amongst both genders.

**TABLE 6:** Age- and race-adjusted regression coefficients (ß) and *p*-values for the relationship of changes in body composition and changes in health-related fitness parameters.

Variable	Gender	Parameter	SBJ (cm)		BAH (sec.)		SAR (cm)		SUP		VO_2_max	
			ß	*p*	ß	*p*	ß	*p*	ß	*p*	ß	*p*
BMI	Boys	Crude	−0.011	0.057	−0.037	0.38	−0.09	0.138	−0.04	0.198	−0.229	0.001
		Adjusted	−0.012	0.56	−0.03	0.51	−0.10	0.155	−0.049	0.163	−0.226	0.002
	Girls	Crude	−0.012	0.05	−0.075	0.002	−0.015	0.72	−0.01	0.696	−0.19	0.000
		Adjusted	−0.12	0.068	−0.08	0.001	−0.008	0.84	−0.01	0.694	−0.197	0.000
%BF	Boys	Crude	−0.03	0.007	−0.229	0.006	0.15	0.566	−0.31	0.000	−.081	0.000
		Adjusted	−0.03	0.01	−0.227	0.01	0.13	0.34	−0.32	0.000	−0.82	0.000
	Girls	Crude	−0.056	0.000	−0.335	0.000	0.03	0.756	−0.22	0.000	−0.899	0.000
		Adjusted	−0.058	0.000	−0.334	0.000	0.03	0.71	−0.23	0.000	−0.88	0.000
WHtR	Boys	Crude	0.00	0.009	0.00	0.34	−0.070	0.589	−0.001	0.067	−0.02	0.00
		Adjusted	0.00	0.008	0.00	0.47	−0.071	0.602	−0.001	0.05	−0.003	0.001
	Girls	Crude	0.00	0.02	−0.001	0.00	−0.070	0.41	0.000	0.44	−0.002	0.00
		Adjusted	0.00	0.028	−0.001	0.00	−0.056	0.52	0.000	0.49	−0.002	0.00

BMI, body mass index; %BF, percentage body fat; WHtR, waist-to-height ratio; SBJ, standing broad jump; BAH, bent arm hang; SAR, sit-and-reach; SU, sit-up; VO_2_max, maximal oxygen uptake.

## Discussion

The present study was carried out to examine a two-year longitudinal development in health-related physical fitness, PA and body composition in randomly selected South African schoolchildren. In general, the results showed a steady decrease in underweight on the one hand, whilst on the other hand a relative increase in overweight amongst the boys and girls was observed. In a study on health-related fitness and weight status in Hong Kong adolescents, Mak et al.^30^ reported higher body weight characteristics and comparatively poorer age- and gender-specific performance measures than amongst the South African children in the present study. However, our study showed an increasing tendency to be fatter and overweight, as indicated by BMI scores of the South African boys and girls over the 2-year period. As many factors affect body composition, it is important that those factors be identified so that preventative measures can be put in place to address them. The outcome of the negative health effects of underweight and obesity is likely to be the development of diseases that emanate from a sedentary lifestyle such as hypertension, cancer and Type II diabetes^5,31,32,33^ which can considerably reduce children's health-related physical fitness.^34^

In the present study, significantly high inter-correlations were found for BMI and %BF, sum of skinfolds and WHtR over the two testing sessions. This finding is not surprising as studies have consistently shown that, because these variables are all indicators of fatness, positive associations amongst them are to be expected.^35^

It was of particular interest in the present study to examine the longitudinal relationship between body composition and fitness performance measures amongst the South African learners; the results showed that changes in BMI and %BF were inversely associated with health-related physical fitness amongst the boys and girls. Furthermore, significant positive relationships were found between changes in WHtR and SBJ in both genders, with correspondingly low inverse correlations noted between WHtR and BAH in girls, and VO_2_max in both genders. These results are probably linked to the decrease in physical activity/exercise and work capacity amongst the children, which consequently reduced their health-related physical fitness performances.^4^ Furthermore, Shang et al.^36^ reported that overweight and obese youth do not perform well, as was shown by their poor performance in SBJ, and in the 50 m sprint and shuttle run, compared with youths of normal weight. These observations show that explosive strength, cardiorespiratory fitness, speed, agility and muscular capability of the youths are progressively reduced owing to excess fat accumulated in the body,^36^ which becomes an extra load for them to carry in weight-bearing tasks or whilst performing gross motor skills.

Research has shown that physical fitness is another important issue from a public health perspective.^13,37^ According to Brunet et al*.*^38^ and Artero et al.^4^ who examined whether there is a relationship between weight status and health-related physical fitness in youth, there was a decrease in physical fitness with increasing BMI amongst children. Although the major influence of fat mass and fat-free mass is still not clear,^4^ it should be noted that body composition constitutes an important element of health-related physical fitness and is a powerful factor in providing specific information on wellness.^5^

One influence that could account for the tendency for South African children to develop excess BF and weight is the fact that they have limited exposure to PA in school. For instance, it was reported in the study conducted by Reddy et al.^39^ that, amongst South African adolescents, more than 54.3% have physical education (PE) classes as part of their school curriculum, and more than 52.8% engage in vigorous physical activity (PA) during school hours. Despite this shortcoming (i.e. excessive BF amongst learners), attention is not paid to PE in the public school system, and many schools are constructed without playgrounds.^39^ To stem the rising trend of obesity and physical inactivity in South African high school learners, it is of vital importance that the school system provides children and adolescents ample opportunity to be physically active. The need to design PA intervention programmes, even in the temporary absence of PE, is therefore urgent and imperative.

## Limitations of the study

The findings of the present study should be interpreted in the light of the following limitations. In view of the longitudinal nature of the study, only a small sample of schoolchildren was involved. It was also not feasible to involve more children as the sample was based on the permission granted by the authorities of the schools where the study was conducted, and care was taken not to disrupt the learners’ classes as a result of their participation in the research. The results might have been different if a larger sample size had been used. As the study was undertaken in 6 public high schools in the Tlokwe Municipality area of the Dr Kenneth Kaunda District Municipality, the findings should not be generalised as reflecting the status of PA, physical fitness and body composition amongst schoolchildren in Tlokwe Municipality and North-West Province as a whole. Such a generalisation would require much larger samples that would be representative of schools in the province.

## Conclusion and recommendations

Based on the findings, it is concluded that changes in BMI, %BF and WHtR are negatively associated with strength and running performances in children. The relative increase in overweight, especially in girls, negatively affected their endurance running and static strength performances. Based on these findings, we therefore recommend the need for PA interventions in school PE programmes. In addition, future studies that will cover larger samples of South African learners are required.
